# Supplementary material for: Genetic basis of maneb-induced dopaminergic neurodegeneration in Drosophila
Source: G3 (Bethesda). 2025 Jul 11;15(9):jkaf159. doi: 10.1093/g3journal/jkaf159 (PMC12405872; doi:10.1093/g3journal/jkaf159)
Supplement: jkaf159_Supplementary_Data [file jkaf159_supplementary_data.zip › Figure_S1_Legend_G3-2025-406039.docx]

**Figure S1. Correlation between individual DA neuron clusters and total DA neuron count.** Scatterplots for PPM1/2, PPM3 or PPL1 clusters vs. total DA neuron counts shows a strong positive correlation in each case.
